# Efficient conversion of syngas to linear α-olefins by phase-pure χ-Fe_5_C_2_

**DOI:** 10.1038/s41586-024-08078-5

**Published:** 2024-10-16

**Authors:** Peng Wang, Fu-Kuo Chiang, Jiachun Chai, A. Iulian Dugulan, Juan Dong, Wei Chen, Robin J. P. Broos, Bo Feng, Yuanjun Song, Yijun Lv, Quan Lin, Rongming Wang, Ivo A. W. Filot, Zhuowu Men, Emiel J. M. Hensen

**Affiliations:** 1 CTL Technology Research Center, National Institute of Clean-and-Low-Carbon Energy, CHN Energy, Beijing, People’s Republic of China; 2 Laboratory of Inorganic Materials and Catalysis, Department of Chemical Engineering and Chemistry, Eindhoven University of Technology, Eindhoven, The Netherlands; 3 Fundamental Aspects of Materials and Energy Group, Delft University of Technology, Delft, The Netherlands; 4 Data Technology Group, China Energy Investment Group Archives, CHN Energy, Beijing, People’s Republic of China; 5 Beijing Advanced Innovation Center for Materials Genome Engineering, Beijing Key Laboratory for Magneto-Photoelectrical Composite and Interface Science, State Key Laboratory for Advanced Metals and Materials, University of Science and Technology Beijing, Beijing, People’s Republic of China

**Keywords:** Heterogeneous catalysis, Catalysis, Materials for energy and catalysis

## Abstract

Oil has long been the dominant feedstock for producing fuels and chemicals, but coal, natural gas and biomass are increasingly explored alternatives^1–3^. Their conversion first generates syngas, a mixture of CO and H_2_, which is then processed further using Fischer–Tropsch (FT) chemistry. However, although commercial FT technology for fuel production is established, using it to access valuable chemicals remains challenging. A case in point is linear α-olefins (LAOs), which are important chemical intermediates obtained by ethylene oligomerization at present^4–8^. The commercial high-temperature FT process and the FT-to-olefin process under development at present both convert syngas directly to LAOs, but also generate much CO_2_ waste that leads to a low carbon utilization efficiency^9–14^. The efficiency is further compromised by substantially fewer of the converted carbon atoms ending up as valuable C_5_–C_10_ LAOs than are found in the C_2_–C_4_ olefins that dominate the product mixtures^9–14^. Here we show that the use of the original phase-pure χ-iron carbide can minimize these syngas conversion problems: tailored and optimized for the process of FT to LAOs, this catalyst exhibits an activity at 290 °C that is 1–2 orders higher than dedicated FT-to-olefin catalysts can achieve above 320 °C (refs. ^12–15^), is stable for 200 h, and produces desired C_2_–C_10_ LAOs and unwanted CO_2_ with carbon-based selectivities of 51% and 9% under industrially relevant conditions. This higher catalytic performance, persisting over a wide temperature range (250–320 °C), demonstrates the potential of the system for developing a practically relevant technology.

## Abstract (web summary)

When used in its phase-pure form, iron carbide catalyses the conversion of syngas to linear α-olefins with high activity and selectivity under industrially relevant conditions.

## Main

With iron carbide as the active phase in iron-based FT catalysts, earlier work has explored the stability of different carbide phases^16–18^ and shown that phase-pure ε-iron carbide does not produce CO_2_ as a primary product unlike typical iron-based FT catalysts^16^. Informed by this and aiming for an active phase that can be operated in a stable manner in a broad temperature range required for optimization of the FT-to-LAO (FTLAO) process, we focused on obtaining phase-pure χ-iron carbide^16–18^. Different from the procedure to obtain pure ε-iron carbide, passivation of fully reduced Raney iron before carburization in syngas yielded phase-pure χ-iron carbide without competing iron oxide phases.

Table 1 compares the FTLAO performance of our χ-iron carbide (χ-Fe_5_C_2_) against that of other catalysts, which illustrates the high activity of our system. Already at 250 °C, the CO conversion time yield (CTY) is 3–7 times higher than that of all the state-of-the-art catalysts from the literature^13–15,19,20^, which were typically evaluated at substantially higher reaction temperatures above 320 °C. At 250 °C, we also observe a low CO_2_ selectivity of 11% and associated high overall carbon efficiency (selectivity to hydrocarbons and oxygenates) of 89%. Table 1 shows that with few exceptions, the reference catalysts exhibit a much higher CO_2_ selectivity ranging from 37% to 47% and a lower carbon efficiency ranging from 63% to 53% (refs. ^13,14,19^). Despite the low CO_2_ selectivity of 13% obtained with a hydrophobic FeMn@Si catalyst, this system shows a lower activity, even at a much higher reaction temperature of 320 °C. Although more expensive catalysts based on cobalt carbide are active in the same low-temperature range as our catalyst, typical CTY values are still substantially lower at an unfavourable CO_2_ selectivity close to 50% (ref. ^19^). In terms of CO_2_ selectivity, Co_1_Mn_3_–Na_2_S exhibits a notably low value of less than 3% at 240 °C that, however, increases rapidly as the temperature is ramped up to improve the activity^20^. We also note that, as discussed in the literature^21,22^, operation at elevated temperatures shifts the product distribution of cobalt-based catalysts towards less desired products and renders them more susceptible to poisoning.

**Table 1:** Catalytic performance comparison of FTLAO and reported catalysts

Sample	*T* (°C)	CO conversion (%)	Carbon efficiency^a^ (%)	*P* (MPa)^b^	SV (ml ${{\bf{g}}}_{{\bf{cat}}}^{-{\bf{1}}}\,{{\bf{h}}}^{-{\bf{1}}}$)^c^					O/P ratio^d^	Carbon-based selectivity (%)		Catalyst time yield (μmol ${{\bf{g}}}_{{\bf{cat}}}^{-{\bf{1}}}\,{{\bf{s}}}^{-{\bf{1}}}$)	
					H_2_	CO	Inert gas	Total	H_2_/CO ratio		CO_2_	Target olefins^e^	CO	Target olefins^e^
χ-Fe_5_C_2_ (this work)	250	27.8	88.8	2.3	12,000	8,000	8,000	28,000	1.5	1.2	11.2	24.3	27.6	6.7
Mn-χ-Fe_5_C_2_ (this work)	250	16.0	90.7	2.3	12,000	8,000	8,000	28,000	1.5	4.1	9.3	50.7	15.9	8.0
Mn-χ-Fe_5_C_2_ (this work)	250	46.1	90.6	3.0	3,000	1,900	100	5,000	1.5	4.1	9.4	48.5	10.9	5.3
Mn-χ-Fe_5_C_2_ (this work)	290	70.7	78.1	2.5	18,000	11,400	600	30,000	1.5	4.5	21.9	42.0	100.0	42.0
Mn-χ-Fe_5_C_2_ (this work)	290	52.8	79.2	2.5	36,000	22,800	1,200	60,000	1.5	4.6	20.8	43.6	149.3	65.1
Mn-χ-Fe_5_C_2_ (this work)	320	91.2	68.0	2.5	36,000	22,800	1,200	60,000	1.5	2.7	32.0	28.5	257.9	73.5
Na_2_S-Fe-CNF (ref. ^13^)	340	88.0	58.0	2.0	328.5	328.5	73	730	1.0	4.3	42.0	30.2	3.6	1.1
FeMn@Si (ref. ^15^)	320	56.1	87.0	2.0	2,533	1,267	200	4,000	2.0	5.1	13.0	44.0	8.8	3.9
Mn-Na-Co_2_C (ref. ^19^)	250	28.6	53.4	1.0	647	1,293	60	2,000	0.5	5.6	46.6	17.0	4.6	0.8
Co_1_Mn_3_–Na_2_S (ref. ^20^)	240	18	>97	1.0	4,800	2,400	800	8,000	2.0	4.3	<3	29.0	5.4	1.6
	280	20	79.0	1.0	4,800	2,400	800	8,000	2.0	2.0	21.0	17.4	6.0	1.0
Fe-Al_2_O_3_ (SCS350)^f^ (ref. ^14^)	320	22.1	63.3	1.5	3,000	3,000	–	6,000	1.0	0.4	36.7	9.1	8.2	0.7
Fe-Al_2_O_3_ (SAP)^f^ (ref. ^14^)	436	21.4	63.3	1.5	3,000	3,000	–	6,000	1.0	1.3	36.7	11.6	8.0	0.9
Fe-Al_2_O_3_ (SCP)^f^ (ref. ^14^)	500	18.3	58.9	1.5	3,000	3,000	–	6,000	1.0	2.7	41.1	25.4	6.8	1.7

The data shown are for a fixed-bed reactor; CTY represents the number of moles of converted CO or moles of produced target olefins per gram of catalyst per second; carbon efficiency is the fraction of C atoms from CO converted ending up in hydrocarbons and oxygenates.

^a^Overall carbon-based selectivity of hydrocarbon and oxygenate products.

^b^Reaction pressure.

^c^Syngas (H_2_ + CO) SV.

^d^For Mn-χ-Fe_5_C_2_, χ-Fe_5_C_2_ and FeMn@Si, the value refers to the O/P ratio in C_2_–C_10_ products. For other samples, it refers to the O/P ratio in C_2_–C_4_ products.

^e^For Mn-χ-Fe_5_C_2_, χ-Fe_5_C_2_ and FeMn@Si, target olefins are C_2_–C_10_ LAOs. For other samples, target olefins are light olefins (C_2_–C_4_).

^f^SCS350, SAP and SCP are the catalyst sample codes used in ref. ^14^.

**Extended Data Fig. 1:**
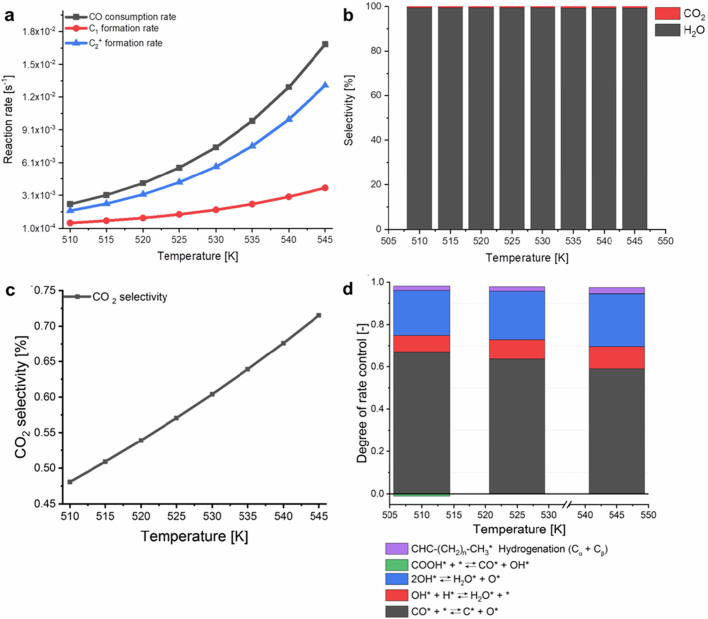
Microkinetics simulations for χ-Fe_5_C_2_-(100) surface. (a) CO consumption (grey), C_1_ formation (red), and C_2+_ formation (blue) rates as a function of the reaction temperature (0.1 MPa, H_2_/CO = 2). Rates are determined by microkinetics simulations based on DFT-computed reaction energetics. (b) Selectivity towards H_2_O and CO_2_ as a function of the reaction temperature (0.1 MPa, H_2_/CO = 2). (c) CO_2_ selectivity of the FT reaction between 510 K and 545 K at 1 bar in the zero-conversion limit. (d) Degree of rate control analysis in microkinetic simulations at 510 K, 525 K and 545 K (0.1 MPa, H_2_/CO = 2). Rates are determined by microkinetics simulations based on DFT-computed reaction energetics.

**Extended Data Fig. 2:**
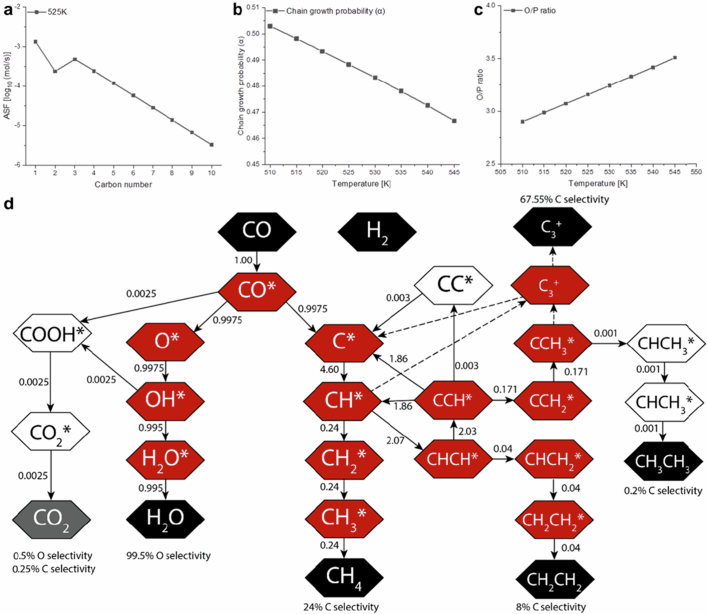
Microkinetics simulations of hydrocarbon products for χ-Fe_5_C_2_-(100) surface. (a) Distribution of hydrocarbons obtained during microkinetics simulations of the FT reaction at 525 K. (b) the chain-growth probability α and (c) the olefins-to-paraffins (O/P) ratio as a function of the reaction temperature (0.1 MPa, H_2_/CO = 2). (d) Network plot at 525 K: the net relative rates (CO conversion rate set at unity) are indicated next to the arrows. The dashed arrows indicate the routes towards C_3_^+^ formation (0.1 MPa, H_2_/CO = 2). Red hexagons represent the major surface intermediates, black hexagons the major gaseous components, grey hexagons the minor gaseous components, and white hexagons minor surface intermediates. Surface intermediates and products with a relative contribution less than 10^−4^ were omitted. CO dissociation proceeds via direct C-O bond scission and the resulting O atom is mainly removed as H_2_O. The dominant C-C coupling mechanism involves association of two CH species to produce the C_2_ intermediate and association of CH and CR for obtaining higher hydrocarbons.

We used density functional theory calculations and microkinetics simulations to explore FT chemistry as catalysed by χ-Fe_5_C_2_ (see Methods, Extended Data Figs. 1 and 2 and Extended Data Table 1 for full details), finding the expected exponential increase in CO conversion and hydrocarbon formation rates with temperature and a C_2+_ hydrocarbon distribution according to Anderson–Schulz–Flory (ASF) theory (Extended Data Figs. 1a and 2a). Simulated olefin-to-paraffin (O/P) ratios are in the same range as the experimentally observed ratios and exhibit a minor dependence on temperature (Extended Data Fig. 2c), indicating that both olefins and paraffins are primary products. The calculations also show that for χ-Fe_5_C_2_, the energy barrier for H_2_O formation is lower than that for CO_2_ formation and that oxygen from CO dissociation will thus be removed primarily as water, which explains the low CO_2_ selectivity of our system. This observation also explains the importance of using a phase-pure catalyst: incomplete conversion of the catalyst precursor to χ-Fe_5_C_2_ will result in the presence of competing phases that often include iron oxides, which are known as good catalysts for the water–gas shift reaction that generates CO_2_ from CO and water^23^.

**Extended Data Fig. 3:**
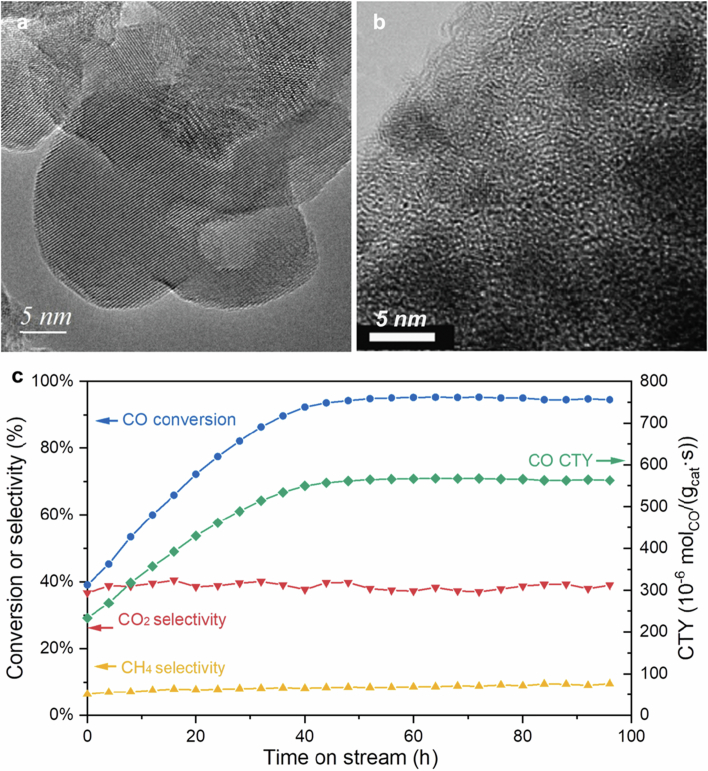
Comparison between phase-pure χ-Fe-carbide and non-phase-pure χ-Fe-carbide. (a)-(b) Comparison representative HRTEM images: (a) phase-pure χ-Fe-carbide in the present work and (b) non-phase-pure χ-Fe-carbide catalyst (38-Fe@C) from reference^24^. (c) Catalytic performance of 1 wt% K-promoted phase-pure χ-Fe-carbide catalyst. Reaction condition: 325 °C, 2.3 MPa, SV H_2_/CO/He = 72,000/48,000/48,000 ml/(g_cat_∙h). Micrograph in **b** adapted from ref. ^24^, Springer Nature Limited.

High-resolution transmission electron microscopy (HRTEM) imaging indicates that very little amorphous carbon is deposited on the phase-pure χ-iron carbide, which contributes to its high and stable CO conversion rate and low CO_2_ selectivity (Extended Data Fig. 3a). This contrasts with the substantial level of carbon deposition onto the surface of a catalyst containing 86% χ-Fe_5_C_2_ and iron oxide phases (Extended Data Fig. 3b), which was reported^24^ to reach a high CO CTY of 303 μmol ${{\rm{g}}}_{{\rm{cat}}}^{-1}\,{{\rm{s}}}^{-1}$ but also a high CO_2_ selectivity of 45% at a temperature of 340 °C. As this catalyst used potassium^24^ as a promoter known to improve CO conversion, we also promoted our phase-pure χ-Fe_5_C_2_ with potassium and achieved at a lower temperature of 325 °C a higher CO CTY of 570 μmol (${{\rm{g}}}_{{\rm{cat}}}^{-1}\,{{\rm{s}}}^{-1}$), lower CO_2_ selectivity of 37% and better stability (Extended Data Fig. 3c). Our phase-pure χ-Fe_5_C_2_ catalyst thus outperforms the phase-impure χ-Fe_5_C_2_ catalyst in terms of activity and CO_2_ selectivity, although the better performance will also in part be due to differences in catalyst particle size (about 17 nm for our catalyst, see Extended Data Fig. 7i, versus about 3 nm reported^24^ for the phase-impure catalyst)^25^. To achieve a high carbon efficiency and high LAO yields, we opted to not use potassium promoters that inevitably increase the CO_2_ selectivity.

**Extended Data Fig. 4:**
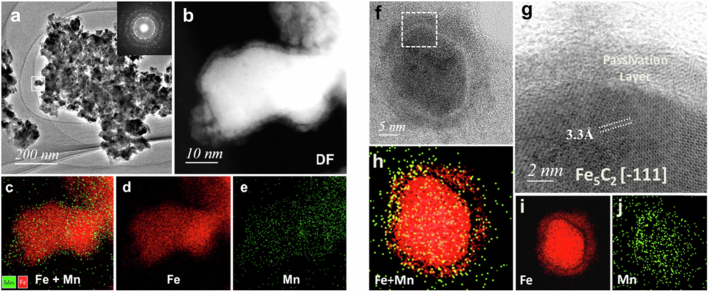
Ex situ TEM images of Mn-χ-Fe_5_C_2_ and used Mn-χ-Fe_5_C_2_. (a) Representative TEM image of Mn-χ-Fe_5_C_2_. The selected-area electron diffraction (SAED) pattern in the inset shows the diffraction pattern of χ-Fe_5_C_2_. (b) Dark-field (DF) image of a single particle of χ-Fe_5_C_2_ (white-square in a) and (c)-(e) corresponding STEM-EDS images, showing the uniform distribution of Mn promoter on the χ-Fe_5_C_2_ surface. (f)-(j) Mn-χ-Fe_5_C_2_ after use in the FT reaction at 290 °C, 2.5 MPa, SV H_2_/CO/Ar = 18,000/11,400/600 ml/(g_cat_∙h), 160 h. (f) Bright-field (BF) image of a single particle of χ-Fe_5_C_2_. (g) BF image of a single χ-Fe_5_C_2_ particle (white-square in f) and (h)-(j) corresponding STEM-EDS images showing the distribution of Mn promoter on the surface of used Mn-χ-Fe_5_C_2_. The Mn promoter content is 10 wt%.

**Extended Data Fig. 5:**
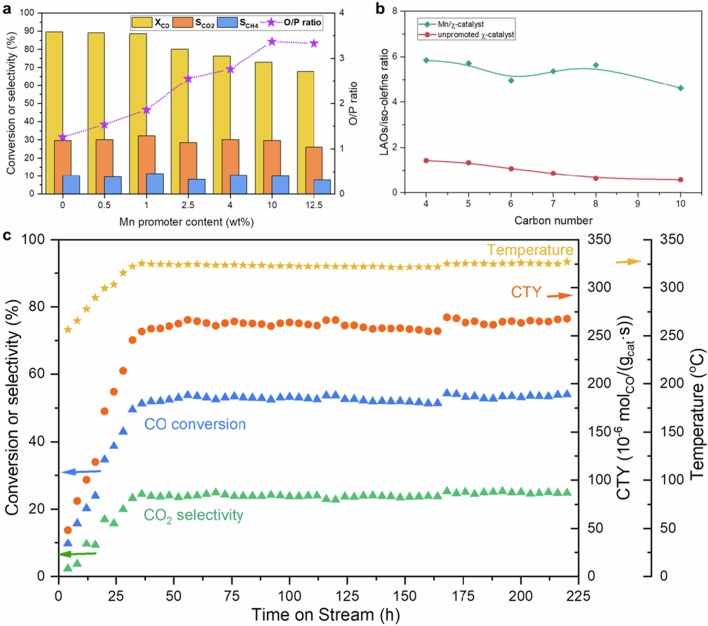
Catalytic activity tests for Mn-promoted FTLAO catalysts. (a) Catalytic performance of Mn-promoted FTLAO catalysts (Mn-χ-Fe_5_C_2_). Reaction conditions: 315 °C, 2.3 MPa, SV H_2_/CO/He = 36,000/24,000/24,000 ml/(g_cat_∙h). (b) LAOs to iso-olefins ratio as a function of the carbon number for Mn-χ-Fe_5_C_2_ and χ-Fe_5_C_2_. Reaction conditions: 250 °C, 2.3 MPa, SV H_2_/CO/He = 12,000/8,000/8,000 ml/(g_cat_∙h). (c) Stability evaluation for the Mn-χ-Fe_5_C_2_ catalyst. Reaction conditions: 320–325 °C, 2.3 MPa, SV H_2_/CO/He = 60,000/40,000/40,000 ml/(g_cat_∙h). CTY means the catalyst time yield. For (b) and (c), the Mn promoter content is 4 wt%.

However, despite the competitive performance, the O/P ratio and the fraction of LAOs among total olefins obtained with the phase-pure χ-Fe_5_C_2_ catalyst are still relatively low and limit the carbon-based selectivity towards desirable C_2_–C_10_ LAOs to 25%. Mechanistically, this is attributed to olefins, which are the primary products of the FT reaction, re-adsorbing and undergoing hydrogenation and isomerization reactions that convert them into less valuable paraffins and iso-olefins. As the addition of manganese is known to improve the activity and product selectivity of iron-based FT catalysts^26^, we promoted the phase-pure χ-Fe_5_C_2_ catalyst with manganese (Mn-χ-Fe_5_C_2_) and achieved the highest O/P ratio and lowest CO_2_ selectivity at an optimized manganese content of 10% by weight (Extended Data Figs. 4a–e and 5a). Although blocking of active sites by manganese slightly lowers the CO CTY for Mn-χ-Fe_5_C_2_, the promoted catalyst still outperforms all reported catalysts and maintains its high phase purity and associated low CO_2_ selectivity while exhibiting a much improved product distribution (entries 1–3 of Table 1): the O/P ratio of C_2_–C_10_ olefins and the selectivity to target LAOs increase from 1.2 to 4.1 and 24.3% to 50.7%, respectively. In analogy with the manganese promotion effect seen in cobalt-based FT catalysts^26^, these changes are probably due to the stronger CO adsorption compared with H_2_.

The FT product distribution is typically strongly affected by the CO conversion, with higher conversions increasing the CO_2_ selectivity and decreasing the olefin selectivity and O/P ratio^27,28^. This trend is attributed to the water–gas shift reaction involving initially formed water and hydrogenation of initially formed olefins, with both processes becoming more pronounced as the residence time of reactants and therefore also of initial products in the reactor increases to enable higher CO conversions. Extended Data Table 2 illustrates this by showing how the CO_2_ selectivity of the Mn-χ-Fe_5_C_2_ catalyst (manganese-promoted phase-pure χ-Fe_5_C_2_) varies with CO conversion, which depends on temperature and the flow rate of reactants through the reactor.

We next explored the performance of Mn-χ-Fe_5_C_2_ under a range of reaction conditions relevant to industrial practice by varying the temperature between 250 °C and 320 °C and the space velocity (SV; the rate at which reactants are fed into the reactor) between 5,000 and 60,000 ml ${{\rm{g}}}_{{\rm{cat}}}^{-1}\,{{\rm{h}}}^{-1}$) while keeping the pressures at 2.5–3.0 MPa and the H_2_/CO ratio at 1.5 (Table 1, rows 3–6). Table 1 provides an overview of performance data of the top-performing catalyst systems for converting syngas to olefins (and thus excludes potassium-promoted systems). Various reactions conditions (with respect to temperature, pressure, H_2_/CO ratio and contact time) were selected for the systems listed, to ensure the best results in terms of olefin yield. The comparison shows that our Mn-χ-Fe_5_C_2_ catalyst outperforms other catalysts reported in the literature in terms of CO conversion and a low CO_2_ selectivity that implies a high selectivity to desired LAOs. We note, however, that the comparison does not take into account the effect of catalyst particle size differences known to affect^25^ performance, owing to lack of data.

**Fig. 1:**
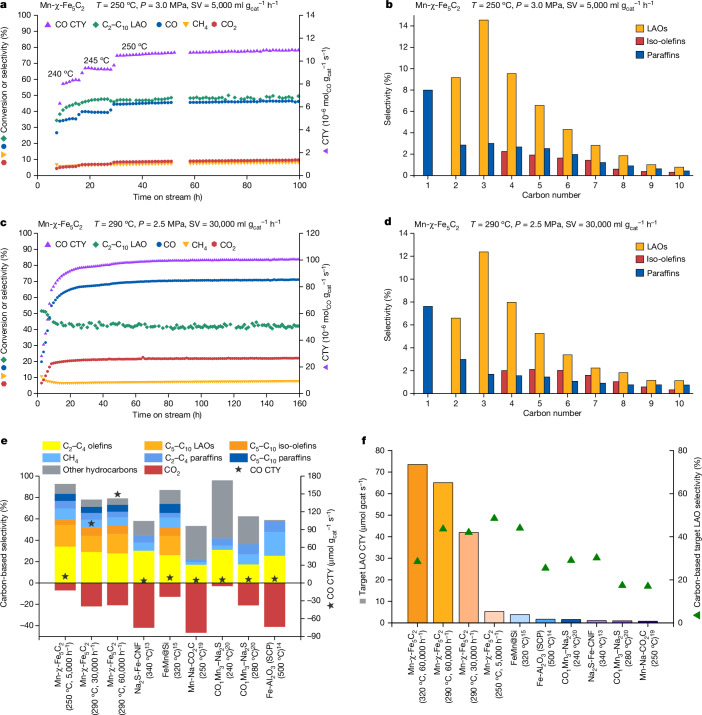
Catalytic performance of optimized Mn-χ-Fe_5_C_2_. **a**,**b**, CO conversion and product selectivity with time on stream (**a**) and averaged product distribution (**b**) at 3.0 MPa, 250 °C and SV H_2_/CO/Ar = 3,000/1,900/100 ml g_cat_ h^−1^. **c**,**d**, CO conversion and product selectivity with time on stream (**c**) and averaged product distribution (**d**) at 2.5 MPa, 290 °C and SV H_2_/CO/Ar = 18,000/11,400/600 ml ${{\rm{g}}}_{{\rm{cat}}}^{-1}\,{{\rm{h}}}^{-1}$. **e**,**f**, Comparison of the distribution of carbon-based products (**e**) and target LAO time yield and carbon-based LAO selectivity (**f**) between Mn-χ-Fe_5_C_2_, χ-Fe_5_C_2_ and reported catalysts from Table 1.

Using a relatively low temperature of 250 °C, a pressure of 3.0 MPa and an SV of 5,000 ml ${{\rm{g}}}_{{\rm{cat}}}^{-1}\,{{\rm{h}}}^{-1}$ (Fig. 1a and Table 1), we achieved a constant CO conversion of about 46% over 100 h with an exceptionally low CO_2_ selectivity of 9.4%, a high carbon-based selectivity towards C_2_–C_10_ LAOs of 48.5% and a CO CTY of 10.9 μmol (${{\rm{g}}}_{{\rm{cat}}}^{-1}\,{{\rm{s}}}^{-1}$). Using a slightly higher but still moderate temperature of 290 °C, a pressure of 2.5 MPa and an SV of 30,000 ml ${{\rm{g}}}_{{\rm{cat}}}^{-1}\,{{\rm{h}}}^{-1}$ (Fig. 1c and Table 1) that resulted in less favourable CO_2_ selectivity (21.9%) and target LAO selectivity (42.0%), we achieved an almost tenfold higher CO CTY of 100.0 μmol ${{\rm{g}}}_{{\rm{cat}}}^{-1}\,{{\rm{s}}}^{-1}$ at a stable and high CO conversion of 70.7%. The average carbon-based selectivities of LAOs, iso-olefins and paraffins under these two operating conditions are shown in Fig. 1b,d. The amount of higher hydrocarbons follows the ASF distribution. The decrease in the ratio of LAOs to iso-olefins with increasing carbon number (Extended Data Fig. 5b) is attributed to consecutive reactions that preferentially convert the heavier products that reside for a longer time in the reactor^29,30^.

An advantage of operating at a moderate temperature is the high CO conversion achieved at a high SV of 60,000 ml ${{\rm{g}}}_{{\rm{cat}}}^{-1}\,{{\rm{h}}}^{-1}$ at 2.5 MPa with a high CTY towards LAOs. The highest CO CTY is achieved at our highest temperature of 320 °C with a pressure of 2.5 MPa and an SV of 60,000 ml ${{\rm{g}}}_{{\rm{cat}}}^{-1}\,{{\rm{h}}}^{-1}$, with CO_2_ selectivity also increasing to 32% but still remaining substantially below values reported in the literature for FT catalysts operated under harsh conditions in Table 1. The increase in CO_2_ selectivity is a natural consequence of high CO conversion levels while using a H_2_/CO feed ratio of 1.5, which will deplete H_2_ and thereby suppress removal of oxygen from CO dissociation in the form of H_2_O and allow CO_2_ formation instead. Although H_2_/CO ratios ranging from 0.5 to 2.0 are common for FT performance evaluations (Table 1), we kept the ratio constant at 1.5 for all of our experiments because a H_2_/CO ratio of between 1 and 2 represents the composition of commercial syngas feeds. A higher H_2_/CO ratio affects the FT performance mainly through higher CO conversion and lower CO_2_ selectivity (due to the competition of oxygen removal as CO_2_ and H_2_O just mentioned^31^). Our phase-pure Mn-χ-Fe_5_C_2_ catalyst exhibits a higher CO conversion and a lower CO_2_ selectivity than those of the top-performing catalysts at a H_2_/CO ratio of 2.0, emphasizing the high performance of our catalyst, which is maintained over a range of operating conditions.

**Extended Data Fig. 6:**
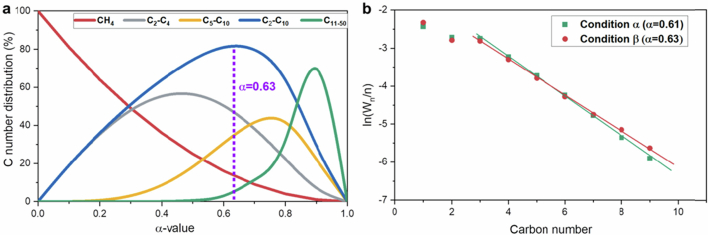
Hydrocarbon product distribution study for FTLAO catalysts. (a) Predicted ASF hydrocarbon product distribution as a function of the chain-growth probability (α). The C_2_-C_10_ products selectivity is maximized when the α value is ~0.63. (b) Experimental ASF plots for Mn-χ-Fe_5_C_2_ obtained under different reaction conditions. W_n_ is the fraction by weight of a hydrocarbon with carbon number n. Reaction conditions: (green) 250 °C, 3.0 MPa, SV H_2_/CO/Ar = 3,000/1,900/100 ml/(g_cat_∙h); (red) 290 °C, 2.5 MPa, SV: H_2_/CO/Ar = 18,000/11,400/600 ml/(g_cat_∙h). The chain-growth probability (α) was experimentally determined by considering C_3_-C_9_ hydrocarbons obtained from the product effluent after 60 h time on stream.

The ASF distribution predicts that a probability *α* of about 0.63 for further growth of a hydrocarbon chain versus termination will yield the highest fraction of C_2_–C_10_ hydrocarbons of 81% (Extended Data Fig. 6a). Careful product analysis (Extended Data Fig. 6b) shows that the experimentally determined values of *α* for our catalyst are close to this optimum: 0.61 and 0.63 at reaction temperatures of 250 and 290 °C, respectively, with corresponding experimentally obtained fractions of C_2_–C_10_ products of 83.1% and 81.5% that are close to the predicted ASF value. The performance of our optimized Mn-χ-Fe_5_C_2_ catalyst is also notable for producing much less CH_4_ than predicted by the ASF distribution and generating substantial amounts of more valuable reaction products. Figure 1e plots the CTY and carbon-based selectivity for Mn-χ-Fe_5_C_2_ and reported catalysts in Table 1, with valuable reaction products as positive contributions and undesirable CO_2_ as the negative one. Mn-χ-Fe_5_C_2_ operated at 250 °C exhibits the highest selectivity to lower olefins (slightly higher than the selectivity reported for Na_2_S-promoted Fe-CNF at 340 °C)^13^ while also generating 20% of products in the form of valuable LAOs. At 290 °C, Mn-χ-Fe_5_C_2_ shows a similar selectivity to C_2_-C_10_ LAOs and a 10–15 times higher CTY compared to that of FeMn@Si (ref. ^15^). The exceptionally low CO_2_ selectivity for our catalyst (9%) is highly advantageous for processes targeting chemicals such as LAOs as the manganese promoter suppresses hydrogenation and thereby increases the O/P ratio of the C_2_–C_10_ products to high values between 4.1 and 4.6. This, in conjunction with the near-optimum *α* value, ensures that Mn-χ-Fe_5_C_2_ realizes a carbon-based selectivity towards desirable LAOs that is close to 50%. Moreover, the optimized Mn-χ-Fe_5_C_2_ catalyst operated at a pressure of 2.5 MPa, an SV of 60,000 ml ${{\rm{g}}}_{{\rm{cat}}}^{-1}\,{{\rm{h}}}^{-1}$ and temperatures of 290 or 320 °C achieves a 10- to nearly 100-fold higher LAO CTY than those of the top-performing catalysts described in the literature and operated at similar or higher reaction temperatures (Fig. 1f).

**Fig. 2:**
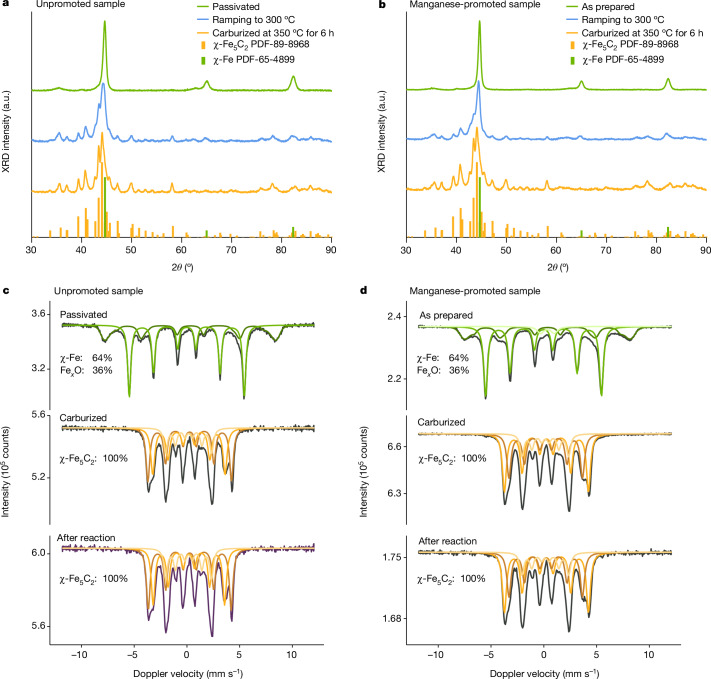
In situ characterization of active-phase formation and evolution. **a**,**b**, In situ XRD patterns of the transformation of unpromoted (**a**) and Mn-promoted (**b**) Raney iron, showing carburization at 300 °C and 350 °C for 6 h (carburization: H_2_/CO = 30, 0.1 MPa, heating from 30 °C to 350 °C at 0.5 °C min^−1^, dwell 6 h). a.u., arbitrary units. **c**,**d**, In situ Mössbauer spectra of the transformation of unpromoted (**c**) and Mn-promoted (**d**) Raney iron, showing as-prepared, after carburization and after FTLAO reaction (carburization: H_2_/CO/He = 100/3.2/21.8, 0.1 MPa, 350 °C for 6 h; FTLAO reaction: H_2_/CO = 1.5 with saturated vapour water (about 0.8 bar) at a total pressure of 2.3 MPa, 265 °C for 12 h). The Mössbauer spectra were acquired at −153 °C.

Given the critical importance of phase purity for the performance of the χ-Fe_5_C_2_ catalyst, we monitored in situ the formation of the phase-pure material from the catalyst precursor pre-treated and activated in the reactor. We used a method differing from that for the preparation of phase-pure ε-FeC_*x*_ (ref. ^16^), in which we passivated Raney iron, after reduction and before carburization, in 1% O_2_ in He at room temperature for 20 h and then carried out carburization into χ-Fe_5_C_2_ by simple exposure to a H_2_/CO/He mixture (100/3.2/21.8) and heating in the reactor (0.5 °C min^−1^, 350 °C, 6 h dwell). The in situ X-ray diffraction (XRD) patterns in Fig. 2a show that χ-Fe_5_C_2_ (Hägg carbide) formation started at 300 °C and was completed after 6 h at 350 °C. Active-phase formation proceeds in the same way in the presence of a manganese promoter (Fig. 2b). This method to prepare phase-pure χ-Fe_5_C_2_ has considerable advantages over methods in the literature^12,13,17,24,32^: it can be carried out in situ by using just a syngas feed with an adjusted H_2_/CO ratio in a single pretreatment step.

**Fig. 3:**
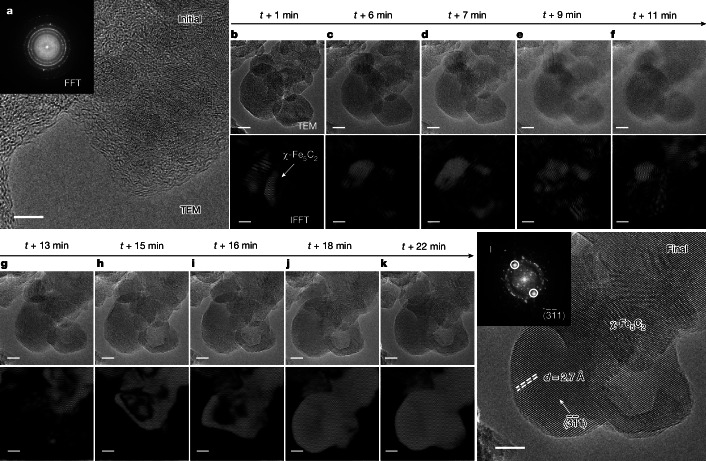
Environmental TEM study of phase-pure χ-Fe_5_C_2_ formation. **a**–**l**, HRTEM images of the transformation from the initial state of Raney iron (**a**), through carburization (**b**–**k**) to the final state (**l**). The dashed lines in **l** mark the lattice spacing of χ-Fe_5_C_2_. The corresponding inverse fast Fourier transform (IFFT) images show the location of χ-Fe_5_C_2_ in real space (environmental TEM conditions: H_2_/CO = 30, 1,200 Pa, 350 °C). FFT, fast Fourier transform. Scale bars, 5 nm.

**Extended Data Fig. 7:**
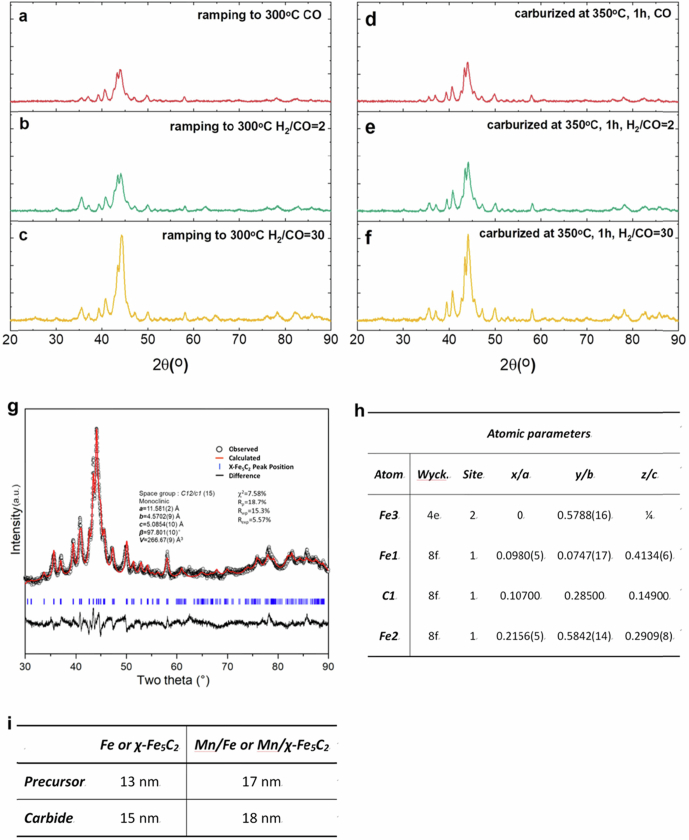
In situ XRD for unpromoted and Mn-promoted Raney Fe. XRD patterns obtained (a)-(c)after ramping to 300 °C in CO, H_2_/CO = 2 and H_2_/CO = 30, 0.1 MPa, (d)-(f), after subsequent carburization at 350 °C for 1 h under CO, H_2_/CO = 2 and H_2_/CO = 30, 0.1 MPa. (g)-(h) Rietveld refinement of χ-Fe_5_C_2_ obtained by the optimized carburization procedure: H_2_/CO = 30, 0.1 MPa, 30 °C to 350 °C at 0.5 °C/min, dwell 6 h. (i) Average crystallites sizes determined by Scherrer analysis of the XRD patterns.

We followed the formation of χ-Fe_5_C_2_ during the carburization process in an environmental transmission electron microscope^33^. HRTEM images and corresponding filtered inverse fast Fourier transform images in Fig. 3 show that the passivated Raney iron is initially present as crystallized iron particles surrounded by an amorphous oxide passivation layer (Fig. 3a). Exposure to syngas at a H_2_/CO ratio of 30 at 1,200 Pa and 350 °C in the environmental transmission electron microscope initiates the χ-Fe_5_C_2_ formation captured by the images shown in Fig. 3b–l. The transformation is seen to start at the inner layers of Raney iron and then extends over the whole region until the final state is reached 30 min later (*t* + 30 min) and a phase-pure χ-Fe_5_C_2_ grain is imaged along the $(\bar{3}\bar{1}1)$ direction with a characteristic lattice spacing of about 2.7 Å (Fig. 3l). Carburization takes place quickly and completes the transformation into χ-Fe_5_C_2_ in less than 0.5 h at a low CO partial pressure of 40 Pa (Supplementary Video 1). This is in line with the in situ XRD data and underpins the efficiency of our approach to preparing χ-Fe_5_C_2_, compared to alternatives that use pure CO or syngas with a much lower H_2_/CO ratio of 2 (Extended Data Fig. 7).

We duplicated the carburization procedure inside a high-pressure Mӧssbauer spectroscopy set-up to monitor in situ the stability of phase-pure χ-Fe_5_C_2_ catalysts under reaction conditions. Figure 2c,d confirms the purity of the χ-Fe_5_C_2_ phase in unpromoted and manganese-promoted catalysts after carburization and subsequent operation under FT reaction conditions. In all samples, Hägg carbide (χ-Fe_5_C_2_) is the only iron phase present (Extended Data Table 3); that is, it is stable during prolonged operation under FTLAO conditions (Fig. 1a,c) at 265 °C. Characterization of a used Mn-χ-Fe_5_C_2_ sample by transmission electron microscopy (TEM) shows a well-maintained χ-Fe_5_C_2_ phase, without signs of carbon deposition or migration of manganese from the iron phase (Extended Data Fig. 4f–j). This is in line with a recent study^34^ showing that manganese suppresses carbon deposition and improves the removal of oxygen, effectively cleaning the surface, which can also explain the high stability we observe. Direct long-term catalytic testing at 320–325 °C, 2.3 MPa and an SV of 100,000 ml_syngas_ ${{\rm{g}}}_{{\rm{cat}}}^{-1}\,{{\rm{h}}}^{-1}$ shows stable Mn-χ-Fe_5_C_2_ performance over 225 h, with a high CO conversion of 53% and a CTY of 265 μmol ${{\rm{g}}}_{{\rm{cat}}}^{-1}\,{{\rm{s}}}^{-1}$ (Extended Data Fig. 5c). From the catalytic data, it follows that the water pressure under these reaction conditions will be at most 0.12 MPa, which is well below the minimum water pressure at which the disadvantageous oxidation of Hägg carbide takes places^35^.

Taken together, our in situ characterization and catalytic data show that phase-pure χ-Fe_5_C_2_ catalysts (both unpromoted and manganese-promoted) are stable and very active for FTLAO. The phase purity of χ-Fe_5_C_2_ enables high CO conversion already at mild conditions and minimizes unwanted CO_2_ production, and manganese promotion inhibits unwanted secondary reactions and thereby contributes to the high selectivity towards desired LAOs. Although industrialization of catalysts inevitably requires addressing process engineering challenges (such as, in this case, catalyst exposure to recycled feed that would include CO_2_ and thereby increase water production), we believe phase-pure χ-Fe_5_C_2_ catalysts will be beneficial in the development of an FTLAO process for converting syngas to valuable LAOs in a competitive manner^9^. Given the demonstrated ability of this type of catalyst to convert CO with high carbon efficiency (that is, low CO_2_ selectivity), we anticipate that it could also benefit other applications that target, for instance, the production of alcohols, aromatics or jet fuels from syngas derived from conventional or renewable carbon feedstock.

## Methods

### Materials

#### Raney iron precursor

Iron-aluminium alloy powder (50:50 by weight, Sigma-Aldrich) was added into an 8 mol l^−1^ KOH (AR, Sinopharm Chemical Reagent) solution in a flask under stirring and heated to 70 °C to dissolve the aluminium in the alloy^36,37^. Afterwards, K^+^ and ${{\rm{AlO}}}_{2}^{-}$ ions were removed by washing with deionized water (ten times) and ethanol (seven times). The iron powder sample was transferred by means of a sealable quartz tube into a glove box and subsequently dried in an argon flow at room temperature for 6 h. The resulting porous iron powder was kept in a container with a seal in a glove box. Before loading the sample in appropriate in situ cells (XRD and Mössbauer spectroscopy characterization) or a stainless-steel reactor for FT activity measurements, the sample was passivated in a flow of 1% O_2_ in helium at room temperature for 20 h.

#### Manganese-Raney iron precursor

Manganese (0.5–12.5 wt%) was added to the Raney iron precursor (directly after removal of aluminium) by wet impregnation using a Mn(NO_3_)_2_ solution. The sample was then dried in a vacuum oven at room temperature for 12 h.

#### Potassium-Raney iron precursor (Extended Data Fig. 3c)

Potassium (1 wt%) was added to the Raney iron precursor (directly after removal of aluminium) by wet impregnation using a KNO_3_ solution. The sample was then dried in a vacuum oven at room temperature for 12 h.

### Characterization

In situ X-ray diffraction (in situ XRD) was carried out on a Rigaku D/max-2600/PC instrument equipped with a D/teX ultrahigh-speed detector and scintillation counter. The X-ray generator consisted of a copper rotating anode with a maximum power of 9 kW. All measurements were carried out at 40 mA and 40 kV. In situ XRD patterns were recorded in an Anton Par XRK-900 cell equipped with a CO/H_2_/Ar gas inlet system.

Environmental TEM (ETEM) images were recorded in an aberration-corrected FEI Titan ETEM G2 instrument at an acceleration voltage of 300 kV (ref. ^38^). Syngas was introduced for 1 h under 1,200 Pa at 320 °C as a pretreatment. The carburization temperature was then raised to 350 °C in about 0.5 h, followed by monitoring the sample continuously.

In situ Mössbauer spectroscopy was carried out in an in situ high-pressure cell suitable for Mössbauer spectroscopy^39^. Transmission ^57^Fe Mössbauer spectra were collected at −153 °C with a sinusoidal velocity spectrometer using a ^57^Co(Rh) source. The source and the sample were kept at the same temperature during the measurements. Mosswinn 4.0 software was used for spectra fitting^40^.

### Catalytic activity measurements

#### In situ carburization of χ-Fe_5_C_2_ and Mn-χ-Fe_5_C_2_ and FTLAO catalytic activity measurements

A 75 mg amount of Raney iron precursor or manganese/Raney iron precursor, the latter also containing 75 mg of Raney iron, was diluted with 1,500 mg silicon carbide and loaded into a stainless-steel tubular fixed-bed reactor with an external diameter of 14.5 mm, internal diameter of 9 mm, length of 305 mm and total internal volume of 20 ml. An exception was the test in Table 1, row 1, for which 225 mg Raney iron of a manganese-Raney iron precursor was diluted with 4,500 mg silicon carbide to achieve a low SV of 5,000 ml ${{\rm{g}}}_{{\rm{cat}}}^{-1}\,{{\rm{h}}}^{-1}$. The catalyst precursor was in situ carburized in a syngas flow (H_2_/CO/He = 100/3.2/21.8) while increasing the temperature to 350 °C at a rate of 1 °C min^−1^, followed by a dwell of 6 h at ambient pressure and an SV of 75,000 ml ${{\rm{g}}}_{{\rm{cat}}}^{-1}\,{{\rm{h}}}^{-1}$. After cooling the reactor to 250 °C (240 °C for the test in Table 1, row 3), the reactor feed was switched to the feed mixture and the reactor pressure was increased to the desired value. The start of the reaction was defined as the time at which these conditions were reached. The reactor was then ramped to the indicated temperature.

For the comparison between χ-Fe_5_C_2_ and Mn-χ-Fe_5_C_2_ (Table 1, rows 1 and 2), the reaction conditions were as follows: a feed mixture SV of H_2_/CO/He = 12,000/8,000/8,000 ml ${{\rm{g}}}_{{\rm{cat}}}^{-1}\,{{\rm{h}}}^{-1}$), a reaction pressure of 2.3 MPa and a reaction temperature of 250 °C. For the evaluation of the Mn-χ-Fe_5_C_2_ catalyst under different temperatures and pressures (Fig. 1a–f and rows 3–6 of Table 1), the reaction conditions were as follows: a feed mixture of H_2_/CO/Ar (internal standard) = 1.5/0.95/0.05 at a total SV of 5,000, 30,000 or 60,000 ml ${{\rm{g}}}_{{\rm{cat}}}^{-1}\,{{\rm{h}}}^{-1}$, a reaction pressure of either 2.5 or 3.0 MPa and a reaction temperature of 250, 290 or 320 °C (heating rate 0.1 °C min^−1^). For the evaluation of the Mn-χ-Fe_5_C_2_ catalyst in Extended Data Table 2, the reaction conditions were as follows: a feed mixture SV of H_2_/CO = 1.5 at a total SV of 30,000, 60,000, 75,000 or 90,000 ml ${{\rm{g}}}_{{\rm{cat}}}^{-1}\,{{\rm{h}}}^{-1}$ with no inert gas added, a reaction pressure of 2.5 MPa and a reaction temperature of 250, 270, 290 or 310 °C (heating rate 0.1 °C min^−1^). For the evaluation of Mn-χ-Fe_5_C_2_ (Extended Data Fig. 5a) and K-χ-Fe_5_C_2_ (Extended Data Fig. 3c), the reaction conditions were as follows: a feed mixture SV of H_2_/CO/He = 36,000/24,000/24,000 ml ${{\rm{g}}}_{{\rm{cat}}}^{-1}\,{{\rm{h}}}^{-1}$ or 72,000/48,000/48,000 ml ${{\rm{g}}}_{{\rm{cat}}}^{-1}\,{{\rm{h}}}^{-1}$ and a reaction pressure at 2.3 MPa. The reaction temperature was 315 or 325 °C for these measurements (heating rate 0.5 °C min^−1^). For the long-term test for Mn-χ-Fe_5_C_2_ (Extended Data Fig. 5c), the reaction conditions were as follows: a feed mixture SV of H_2_/CO/He = 60,000/40,000/40,000 ml ${{\rm{g}}}_{{\rm{cat}}}^{-1}\,{{\rm{h}}}^{-1}$ and a reaction pressure at 2.3 MPa. We used a low ramp rate of 2 °C h^−1^ to avoid overheating issues due to the exothermicity of the reaction. Therefore, it took 35 h to raise the temperature from 250 °C to 320 °C.

The effluent gas flow was analysed by an online Agilent 7890 gas chromatograph equipped with two thermal conductivity detectors and one flame ionization detector. Each result was acquired from a single experiment. All of the SVs are calculated on the basis of the weight of the Raney iron catalyst precursor without a promoter or silicon carbide diluent.

The CO conversion and product selectivity were calculated as below.

The CO conversion (${X}_{{\rm{CO}}}$) was calculated by

1 $${X}_{{\rm{CO}}}=\frac{{{\rm{CO}}}_{{\rm{inlet}}}-{{\rm{CO}}}_{{\rm{outlet}}}}{{{\rm{CO}}}_{{\rm{inlet}}}}\times 100 \% $$

The CO_2_ selectivity (${S}_{{{\rm{CO}}}_{2}}$) was calculated by

2 $${S}_{{{\rm{CO}}}_{2}}=\frac{{{\rm{CO}}}_{{2}_{{\rm{outlet}}}}}{{{\rm{CO}}}_{{\rm{inlet}}}-{{\rm{CO}}}_{{\rm{outlet}}}}\times 100 \% $$

The carbon-based hydrocarbon selectivity (${S}_{{{\rm{C}}}_{x}{{\rm{H}}}_{y}}$) was calculated by

3 $${S}_{{{\rm{C}}}_{x}{{\rm{H}}}_{y}}=\frac{{x{{\rm{C}}}_{x}{{\rm{H}}}_{y}}_{{\rm{outlet}}}}{{{\rm{CO}}}_{{\rm{inlet}}}-{{\rm{CO}}}_{{\rm{outlet}}}}\times 100 \% $$

The indicated parameters represent the inlet and outlet molar flows determined.

The CO catalyst time yield (${{\rm{CTY}}}_{{\rm{CO}}}$) was calculated by

4 $${{\rm{CTY}}}_{{\rm{CO}}}=\frac{{{X}_{{\rm{CO}}}\times {\rm{CO}}}_{{\rm{inlet}}}}{\mathrm{22,400}\,({\rm{ml}}\,{{\rm{mol}}}^{-1})\times \mathrm{3,600}\,({\rm{s}}\,{{\rm{h}}}^{-1})}$$

The carbon-based hydrocarbon catalyst time yield (${{\rm{CTY}}}_{{{\rm{C}}}_{x}{{\rm{H}}}_{y}}$) was calculated by

5 $${{\rm{CTY}}}_{{{\rm{C}}}_{x}{{\rm{H}}}_{y}}={{\rm{CTY}}}_{{\rm{CO}}}\times {S}_{{{\rm{C}}}_{x}{{\rm{H}}}_{y}}$$

The experimental chain-growth probability was calculated by the ASF distribution as follows

6 $${\rm{ln}}\left(\frac{{W}_{n}}{n}\right)=n{\rm{ln}}\alpha +{\rm{ln}}[{\left(1-\alpha \right)}^{2}/{\alpha }]$$

in which *n* is the number of carbon atoms in a particular hydrocarbon product, W_*n*_ is the weight fraction of a product with *n* number of carbon atoms and *α* is the chain-growth probability.

### Theoretical modelling

#### Density functional theory calculations

Density functional theory (DFT) calculations were carried out to obtain the energetics for elementary reaction steps relevant to the FT reaction on χ-Fe_5_C_2_. All spin-polarized DFT calculations were conducted using the projector augmented-wave method and the Perdew–Burke–Ernzerhof functional, as implemented in the Vienna ab initio simulation package code. Solutions of the Kohn–Sham equations were obtained using a basis set of plane waves with a cutoff energy of 400 eV. Sampling of the Brillouin zone was carried out using a 5 × 5 × 1 *k*-point mesh. Higher cutoff energies or a finer Brillouin zone sampling did not lead to substantial energy differences. All atoms were allowed to relax during the optimization of the empty surfaces. We used a 2 × 2 × 1 unit cell for the (100) surface, containing 80 iron and 32 carbon atoms, with a layer thickness of 10.31 Å. A vacuum layer of 15 Å was added perpendicular to the surface to avoid spurious interactions between neighbouring images. Adsorption of atoms and molecules was carried out on the top side of the slab, whereas the lower half was frozen. A dipole correction was carried out for all adsorbed states. Further technical details such as Vienna ab initio simulation package settings for these iron carbide calculations are described elsewhere^23^. The adsorption energies of the gas-phase molecules were determined by subtracting the energies of the empty surface and the free adsorbate from the adsorbed state. The energy of the adsorbate in the gas phase was obtained by placing a molecule at the centre of a 10 × 10 × 10 Å^3^ unit cell, using the Γ-point for *k*-point sampling. Transition states were acquired using the nudged elastic band method^41^. A frequency analysis was carried out to confirm that all transition geometries correspond to a first-order saddle point on the potential energy surface with an imaginary frequency in the direction of the reaction coordinate. The corresponding normal-mode vibrations were also used to calculate the zero-point energy correction. We also corrected the barriers for the migration of fragments after dissociation by considering the energy difference of the geometry directly after dissociation and their most stable adsorption positions at infinite distance.

We carried out DFT calculations to determine the energetics of elementary reaction steps of the conversion of synthesis gas into hydrocarbons (methane, olefins and paraffins) and CO_2_ and H_2_O. The energy barriers and their corresponding pre-exponential factors are listed in Extended Data Table 1. The (100) surface of χ-Fe_5_C_2_ was selected, because this surface is a stable surface termination of Hägg carbide and also allows for facile C–O bond dissociation, which is an essential step in the FT reaction^42^. Extended Data Table 1 shows forward and backward activation energies and the corresponding pre-exponential factors for the consecutive hydrogenation steps of adsorbed carbon to methane and the removal of oxygen as H_2_O and CO_2_. Extended Data Table 1 shows forward and backward activation energies and the corresponding pre-exponential factors for the C–C coupling reactions and the hydrogenation to ethylene and ethane.

#### Microkinetics modelling

For the construction of the microkinetic model of the FT reaction, differential equations for all reaction intermediates on the catalytic surface were constructed using the rate constants of all considered elementary reaction steps. Herein, we assumed that all adsorbates occupy one active site. For adsorption, we assumed that the adsorbate loses one translational degree of freedom in the transition state with respect to the initial state. For desorption, we assumed that the species gains two translational degrees of freedom and three rotational degrees of freedom in the transition state with respect to the initial state. From these two assumptions, the rate of adsorption and desorption are as follows:

7 $${k}_{{\rm{ads}}}=\frac{P\times A}{\sqrt{2{\rm{\pi }}\times m\times {k}_{{\rm{B}}}\times T}}$$

8 $${k}_{{\rm{des}}}=\frac{{k}_{{\rm{B}}}\times {T}^{3}}{{h}^{3}}\times \frac{A\times \left(2{\rm{\pi }}\times m\times {k}_{{\rm{B}}}\right)}{\sigma {\theta }_{{\rm{rot}}}}\times {{\rm{e}}}^{\frac{{E}_{{\rm{des}}}}{RT}}$$

Herein, ${k}_{{\rm{ads}}}$ is the rate constant for the adsorption of the adsorbate, *P* is the pressure in pascals, *A* is surface area in square metres, *m* is the mass of the reactant in kilograms, *k*_B_ is the Boltzmann constant in joules per kelvin, *T* is the temperature in kelvin, ${k}_{{\rm{des}}}$ is the rate constant for the desorption of the adsorbate, *h* is the Planck constant in joules multiplied by seconds, $\sigma$ is the symmetry number, ${\theta }_{{\rm{rot}}}$ the rotational temperature in kelvin, *E*_des_ is the desorption energy in joules per mole, and *R* is the gas constant in joules per kelvin per mole.

The rate constant (*k*) of an elementary reaction step was determined using the Eyring equation, which is defined as follows:

9 $$k=v{\text{exp}}^{\left(\frac{-{E}_{{\rm{act}}}}{{k}_{{\rm{B}}}T}\right)}$$

in which ${E}_{{\rm{act}}}$ is activation energy in joules per mole, *k*_B_ the Boltzmann constant, *T* the temperature in kelvin, and $v$ the pre-exponential factor in the unit of per second. Pre-exponential factors for the forward and backward reactions can be obtained using:

10 $${v}_{{\rm{forward}}}=\frac{{k}_{{\rm{B}}}T}{h}\left(\frac{{q}_{{\rm{vib}}}^{{\rm{TS}}}}{{q}_{{\rm{vib}}}^{{\rm{IS}}}}\right)$$

and

11 $${v}_{{\rm{backward}}}=\frac{{k}_{{\rm{B}}}T}{h}\left(\frac{{q}_{{\rm{vib}}}^{{\rm{TS}}}}{{q}_{{\rm{vib}}}^{{\rm{FS}}}}\right)$$

in which ${v}_{{\rm{forward}}}$ and ${v}_{{\rm{backward}}}$ refer to the pre-exponential factors for the forward and the backward reaction, respectively, *q*_vib_ is the vibrational partition function of the initial state (IS) and the transition state (TS), and *h* is Planck’s constant.

All microkinetic simulations were carried out using the MKMCXX software suite^43^. The set of differential equations were time-integrated using the backward differentiation formula method until a steady-state solution was obtained. From the steady-state coverages, the rates of the individual elementary reactions steps were obtained using a flux analysis, as implemented in the MKMCXX software. To mimic experimental conditions, the pressure was set to 0.1 MPa over a temperature range between 510 K and 545 K. We adopted a continuously stirred tank reactor with ideal mixing using an SV chosen to obtain differential conditions over the whole temperature range. The H_2_/CO ratio was kept constant at 2:1. Chain growth was considered by involving coupling of two CH_*x*_ adsorbates. Chain growth for hydrocarbon chains up to 20 carbon atoms was considered by treating the growing chain as CR, in which R = alkyl chain and considering that barriers for chain growth are independent of chain length. Adsorption energies of C_2_ and C_3_ intermediates were taken into account explicitly, whereas those of hydrocarbon fragments with more than three carbon atoms were taken to be equal to those of C_3_ intermediates. Proper entropy corrections were made depending on the chain length of the hydrocarbons.

The chain-growth probability was determined from the ASF distribution by considering hydrocarbon products containing 1–20 carbon atoms:

12 $${\alpha }=\frac{{r}_{{\rm{p}}}}{{r}_{{\rm{p}}}+{r}_{{\rm{t}}}}\cong \exp \left(\frac{{\rm{d}}{\rm{ln}}{F}_{{{\rm{C}}}_{n}}^{{\rm{out}}}}{{\rm{d}}n}\right)$$

Herein, the chain-growth probability is defined as the rate of propagation (${r}_{{\rm{p}}}$) over the sum of the rates of propagation and termination (${r}_{{\rm{t}}}$). ${F}_{{{\rm{C}}}_{n}}^{{\rm{out}}}$ corresponds to the flow rate of ${C}_{n}$ in the experiment. This involved simulating the corresponding chain-growth probability (*α*) and C_2_ selectivity within the ASF distribution shown in Extended Data Fig. 2.

#### Results of microkinetic simulations

Extended Data Fig. 1a shows the CO conversion rate and formation rates of CH_4_ (C_1_) and longer hydrocarbons (C_2+_) for the (100) surface of χ-Fe_5_C_2_ as a function of temperature. As expected, the rates exponentially increase with temperature owing to the Arrhenius dependence of the reaction rate constants. The CO conversion rate is in the same range as experimentally observed for iron carbide catalysts^42,44^. The selectivity towards CH_4_ is lower than the total selectivity towards other hydrocarbons, which is important because CH_4_ has a much lower value than higher hydrocarbons. Extended Data Fig. 2a shows the hydrocarbon product distribution, which indicates that, except for C_1_ and C_2_, the longer hydrocarbons are statistically distributed according to the ASF theory. The parameter describing this distribution is the chain-growth probability and its temperature dependence is shown in Extended Data Fig. 2b. This parameter reflects the statistical nature of the growth process in which hydrocarbons can either grow by addition of a C_1_ monomer or desorb as a product. The decrease with temperature shows that termination as products has a higher overall activation energy than chain growth. The value of about 0.5 is slightly lower than experimentally observed in our study. Extended Data Fig. 2c shows the simulated O/P ratio to be around 3.0 with a minor dependence on temperature. These data show that both olefins and paraffins are formed as primary products. The O/P values are in the same range as experimentally observed, namely about 1.5 for the unpromoted catalyst and about 3.6 for the manganese-promoted catalyst. Extended Data Fig. 1b demonstrates that oxygen atoms originating from CO dissociation are predominantly removed as H_2_O, instead of CO_2_. These findings provide an explanation for the low CO_2_ selectivity observed in our experimental study in which a pure χ-Fe_5_C_2_ catalyst was used. In other studies in which the catalyst precursor is not completely converted to χ-Fe_5_C_2_, competing phases often include iron oxides, which are known as good catalysts for the (reverse) water–gas shift reaction.

The computational predictions in Extended Data Table 1 show that, on χ-Fe_5_C_2_, the overall barrier for H_2_O formation (155 kJ mol^−1^) is lower than the barrier for CO_2_ formation (181 kJ mol^−1^). This implies that H_2_O formation is preferred on χ-Fe_5_C_2_ over CO_2_ formation as the oxygen removal step. To verify this prediction based on the overall energy barrier under reaction conditions, we used microkinetic simulations based on DFT-based reaction energetics and found that oxygen removal reactions proceed primarily through the formation of H_2_O (99.4% at 525 K) instead of CO_2_ (0.6% at 525 K; Extended Data Fig. 1c). These simulations showing a very low CO_2_ selectivity pertain to the zero-conversion limit and, thus, represent so-called primary CO_2_ production. The microkinetic simulations also provide a deeper insight into the reaction mechanism including the interplay between the surface intermediates. In Extended Data Fig. 1d we show that, in addition to CO dissociation, oxygen removal and carbon hydrogenation (as part of the chain-growth mechanism) control the overall reaction rate. These findings are in keeping with the periodic trends predicted in previous work^43^.

#### Rietveld refinement XRD patterns

The results of Rietveld refinement of the XRD patterns using the Fullprof software are shown in Extended Data Fig. 7g. Rietveld refinement confirmed the phase purity of χ-Fe_5_C_2_ (no substantial contribution of other phases) with a goodness of fit of *χ*^2^ = 7.58%, *R*_p_ = 18.7%, *R*_wp_ = 15.3% and *R*_exp_ = 5.6%. The space group and lattice parameters listed in Extended Data Fig. 7g are in good agreement with published data for χ-Fe_5_C_2_ (ref. ^45^). The atomic positions of the specific sites for the χ-Fe_5_C_2_ structure are listed in Extended Data Fig. 7h. Note that the data were acquired in an in situ XRD reaction chamber under a nitrogen flow after obtaining the χ-Fe_5_C_2_ particles through the described synthesis method.

## Online content

Any methods, additional references, Nature Portfolio reporting summaries, source data, extended data, supplementary information, acknowledgements, peer review information; details of author contributions and competing interests; and statements of data and code availability are available at 10.1038/s41586-024-08078-5.

## Supplementary information

Supplementary Video 1 In situ observation of phase-pure χ-Fe_5_C_2_ formation by ETEM. HRTEM (left screen), FFT (middle screen) and IFFT images of χ-Fe_5_C_2_ (right screen) show the formation of the χ-Fe_5_C_2_ catalyst under the following ETEM conditions: H_2_/CO = 30, 1,200 Pa, 350 °C.

## Figures and Tables

**Extended Data Table 1:**
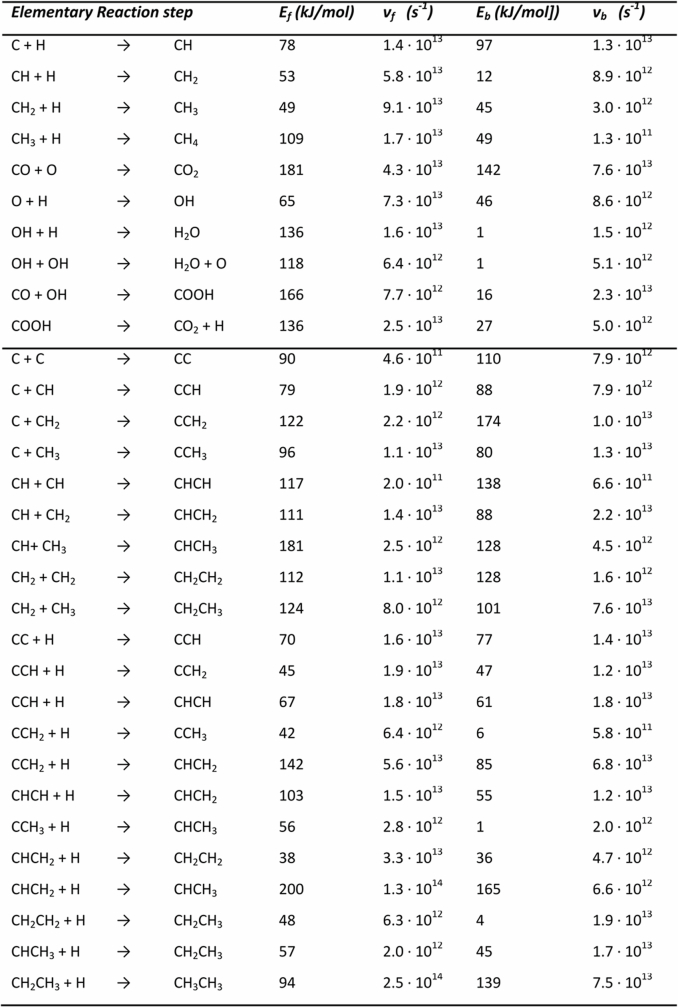
DFT-computed energetics of FT reaction steps for χ-Fe_5_C_2_(100)

Activation energies and pre-exponential factors for methane formation, oxygen removal reactions, ethylene and ethane formation.

**Extended Data Table 2:**
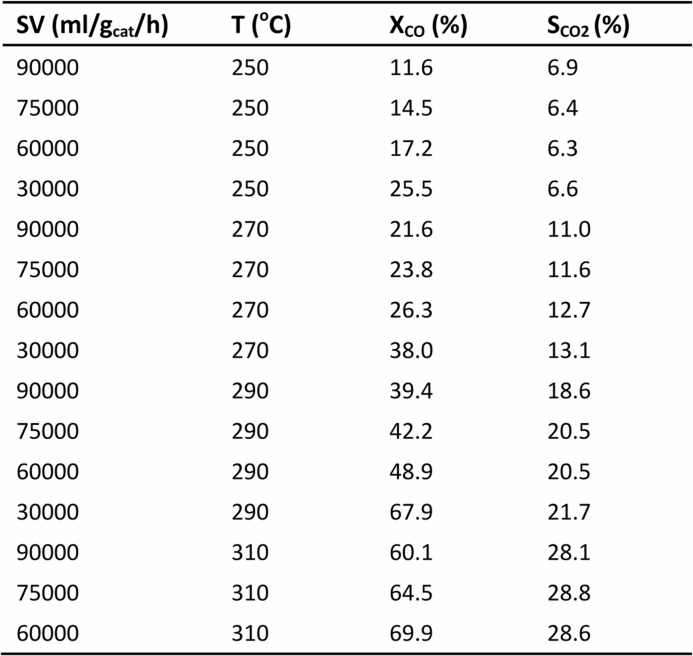
Kinetic study for Mn-promoted FTLAO catalyst

CO conversion and CO_2_ selectivity for Mn-χ-Fe_5_C_2_ catalyst under various reaction conditions (fixed-bed reactor, pressure 2.5 MPa, H_2_/CO = 1.5, no diluent gas added). The Mn promoter content is 10 wt%.

**Extended Data Table 3:**
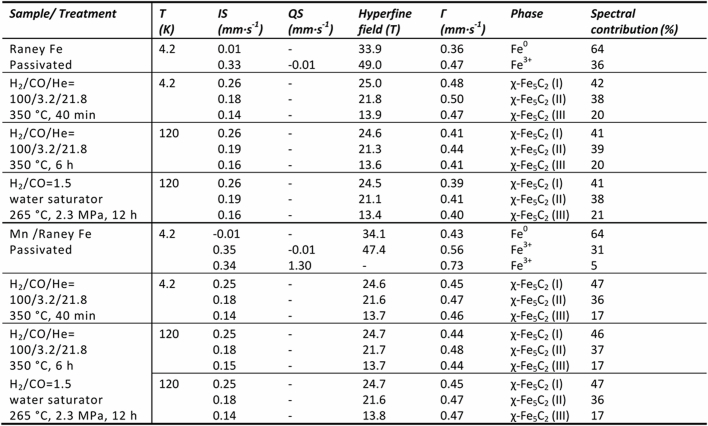
In situ Mössbauer spectra for FTLAO catalysts

Fitting results of in situ Mössbauer spectra of the transformation of unpromoted and Mn-promoted Raney Fe to phase-pure χ-Fe_5_C_2_.

Experimental uncertainties: Isomer shift: I.S. ± 0.02 mm s^−1^; Quadrupole splitting: Q.S. ± 0.02 mm s^−1^; Line width: Γ ± 0.03 mm s^−1^; Hyperfine field: ± 0.1 T; Spectral contribution: ± 3%.

## Data Availability

All data are available in the main text or the Supplementary Information and are also available from the corresponding authors on reasonable request.
